# The Works of Sir James Y. Simpson, Bart., M. D., D. C. L. Volume II

**Published:** 1872-03

**Authors:** 


*Anæsthesia, Hospitalism, Hermaphroditism, and a proposal to stamp out Small Pox and other Contagious Diseases.* By Sir JAMES Y. SIMPSON, Bart., M. D., D. C. L. Edited by Sir W. G. SIMPSON, Bart., B. A. New York: D. Appleton & Co., 1872.

The first half of this work is occupied with an exceedingly interesting history of the discovery and use of Anæsthetics which will be of exceeding interest to those of the profession who are curious on the subject, and is ably treated by Prof. Simpson. The views of Prof. Simpson on Hospitalism form a series of interesting papers, which, however, are perhaps not quite as complete as their author would have desired to make them had life been spared. Hermaphroditism, and a proposal to stamp out Small Pox and other contagious diseases serve to complete one of the volumes of a very interesting and valuable collection of the writings of the late Prof. Simpson.

